# Mobile phone use before and during the COVID-19 pandemic – a panel study of older adults in seven countries

**DOI:** 10.1177/20501579231185479

**Published:** 2023-07-10

**Authors:** Sakari Taipale, Tomi Oinas, Loredana Ivan, Dennis Rosenberg

**Affiliations:** Department of Social Sciences and Philosophy, 4168 University of Jyvaskyla, Finland; Communication Department, National University of Political Studies and Public Administration, Romania; Department of Human Services, 26748 University of Haifa, Israel; Department of Social Sciences and Philosophy, 4168 University of Jyvaskyla, Finland

**Keywords:** aging, digital divide, internet users, media displacement, mobile phone, functionalities, technology use changes

## Abstract

The aim of this study was to investigate the changes in older adults’ mobile phone use from before to during the COVID-19 pandemic. The media displacement and digital divide approaches served as the theoretical frameworks of the study. The data were drawn from the 2018 and 2020 waves of the Aging + Communication + Technology cross-national longitudinal panel study. The sample consisted of older Internet users, aged 62 to 96 (in 2018), from Austria, Canada, Finland, Israel, the Netherlands, Romania, and Spain, who participated in both waves (N = 4,398). Latent class analysis and latent transition analysis with multinomial regression models were the main methods applied to the data. With regard to the findings, three mobile phone function use profiles—Narrow Use, Medium Use, and Broad Use—were identified from the data. Lower age, being married, higher income, and place of residence (in 2018) predicted belonging to the three profiles, while country differences in the prevalence of the profiles were substantial. Between 2018 and 2020, transition from one profile to another was relatively rare but typically toward the “Broad Use” category. Profile transitions were most common in Romania, while stability was highest in Finland, Israel, and Canada. In addition, gender, age, marital status, and place of residence predicted the likelihood of changing from one profile to another between 2018 and 2020. The results suggest that older adults’ mobile phone function use is relatively stable over a two-year time span. While new mobile phone functions are adopted, they seem to augment the spectrum of mobile usage rather than displace older similar functionalities. In addition, demographic, socioeconomic, and country-level digital divides, although slightly modified over time, remain significant among older adults.

## Introduction

Older adulthood is a life stage encompassing significant transitions, such as getting retired, becoming a grandparent, and losing loved ones. Considering that older adults are a heterogenous group (Fernández-Ardèvol et al., 2017; Hänninen et al., 2021), these transitions are accompanied by a range of age-related factors that can either impede or facilitate the use of new mobile technologies. Later-life developments such as shrinking of social network size or deterioration of health are often associated with difficulties in the use of technologies that require dexterity, good eyesight, and continuous learning of new skills (Cavapozzi & Dal Bianco, 2022; Friemel, 2016; Petrovčič et al., 2018). However, in addition to these impeding factors, recent research underscores the proactive role of older technology users who tend to seek information, obtain support, and resolve problems online by taking advantage of mobile media and communication technology (Caliandro et al., 2021a; Hänninen et al., 2021; Neven & Peine, 2017). The outbreak of the COVID-19 pandemic in early 2020 also revealed some unexpected positive developments in older adults’ media and communication technology use. While much research has addressed the benefits of using various social networking sites during the pandemic (e.g., Casanova et al., 2021; Melis et al., 2022; Rolandi et al., 2020), the overall use of smartphones increased as well. For instance, in Finland, the share of people aged 75–89 who owned a smartphone increased considerably from 27% to 42% between 2019 and 2021 (Official Statistics of Finland, 2022). Even though older adults developed their media habits in nondigital media landscapes and persisted in their use of traditional or legacy media (Westlund & Ghersetti, 2015), the pandemic demonstrated that significant changes in mobile communication behavior can also take place in later life.

The aim of this study is to investigate the changes in older adults’ mobile phone use between 2018 and 2020. The two-year observation period began at the time before the COVID-19 pandemic and ended at the point when the toughest COVID-19 restrictions were already lifted in most countries. In particular, the study addresses the effect of demographic and socioeconomic factors on the changes in the scope of mobile phone function use, while cross-country differences are a crosscutting theme of the article. The study is based on a cross-national panel survey (N = 4,398) collected from Internet users aged 62 or older (in 2018). Media displacement, media convergence, and digital divide theories are applied for the interpretation of the results. Ultimately, the study responses to the need to track the digital participation of older adults who were considered to be at risk of digital exclusion when the pandemic broke out (e.g., Marston et al., 2020; Seifert, 2020).

## Theory and literature review

### Media displacement, convergence, and the mobile phone as a meta-medium

Changes in media use over time are often addressed in communication research by media displacement theory, which suggests that new forms of digital media decrease the consumption of older forms of digital or nondigital media. The origin of the theory is associated with McCombs (1972), who argued that resources hinder the growth of the media sector. When a certain amount of time or money is spent on one medium, it cannot be used for the other.

The mobile phone is a special case in this respect. Whereas the first mobile phones were only a little more than the landline, the smartphone turned out to be a self-sufficient meta-medium, accommodating multiple previously separate media, such as the Internet, radio, press, and television (Fortunati & Taipale, 2014; Humphreys et al., 2013). Due to their multifunctionality, the use of smartphones may vary significantly, depending on the applications, functions, and features utilized (Wang et al., 2016). Therefore, the smartphone challenges the idea that media displacement takes place between separate media devices and outlets. In the case of the smartphone, a change in media use can take place inside the covers of this meta-medium.

In the communication studies literature, two competing approaches to media displacement can be identified (Kim et al., 2020; Lee & Lee, 2015; Newell et al., 2008; Nimrod, 2019). The *functional displacement* approach suggests that one medium does not fully replace the other one, but only some of its functionalities. This approach allows for the coexistence of numerous media outlets and provides an explanation for why some older forms of media do not completely disappear when a new medium is introduced (e.g., Nimrod, 2019). The other approach suggests *symmetrical replacement,* in which the use of one medium comes at the expense of the other. The rationale of this approach is based on temporal constraints; thus, it is alternatively called *time displacement* (e.g., Kayany & Yelsma, 2000; Kim et al., 2020). According to it, the increased time spent on one medium activity inevitably leads to a decrease in the time spent on other media. In some cases, the use of one medium may stimulate the use of others, leading to the simultaneous use of multiple media (Lee & Lee, 2015). In connection to Internet use, Shklovski et al. (2004) referred to this as *the augmentation thesis*.

Previous media displacement studies are typically based on cross-sectional designs that do not allow for a proper investigation of changes in media use, and they assume transitions toward newer forms of media (De Waal & Schoenbach, 2010; Kim et al., 2020; Newell et al., 2008; Taipale, 2013). However, as Anderson (2005) showed, changes in media use are much more diverse than what cross-sectional studies imply. The changes may include adoption (in-transition), dropping out (out-transition), and re-adoption (back-transition), not to forget continuity in use (a lack of change over time). While prior research has almost by rule focused on shifts between distinct media platforms or their co-uses (e.g., De Waal & Schoenbach, 2010; Kim et al., 2020; Lee & Lee 2015; Newell et al., 2008; Taipale et al., 2021), the current study focuses on the changes in use within one meta-medium. The mobile phone as a meta-medium also serves as a good example of media convergence (Jenkins, 2006). The mobile phone does not automatically replace the other forms of media, but the old and the new media inform and shape each other inside the same device (Fortunati & Bakardjieva, 2020).

### Gray digital divide

An uneven distribution of mobile phones and their specific functions can be reflected through the concept of the digital divide (Tsatsou, 2011) on three different levels (Norris, 2001): the global divide refers to inequalities occurring between countries (e.g., the industrialized and developed nations), the social divide alludes to demographic and socioeconomic differences within the countries that separate between “digitally rich” and “digitally poor,” and the democratic divide addresses the differences in the utilization of digital resource for the beneficial outcome in the personal and public spheres. This study addresses the digital divides that occur both between countries (the global divide) and within countries (the social divides), unfolding the complex role of demographic and socioeconomic factors as predictors of changes in older adults’ mobile use.

The digital divides that are particularly characteristic of older adults’ technology use are known as the gray (digital) divide (e.g., Friemel, 2016; Sala et al., 2020). Apart from the availability of technology and digital skills, the concept of the gray divide involves differences in the perceptions of and attitudes toward new technology that are typically shaped by younger generations (Millward, 2003). Aligned with van Dijk's (2005) material access in resources and appropriation theory, prior studies have shown that age-related divides typically intersect with other demographic and socioeconomic factors, such as gender, income, education, and place of residence. For example, Seifert and Schelling (2015) found that smartphone users are more likely to be male, highly educated, younger old adults, more affluent, and more technologically affine. Fernández-Ardèvol and Ivan (2013) found gender differences related to goals of use: women used mobile phones more for extensive conversations, while men used them for microcoordination. In contrast, Friemel's (2016) study analyzing older Swiss Internet users (aged 65 or more) showed that gender differences disappear when controlled for other sociodemographic and socioeconomic factors. However, both education and income, as well as the social context of Internet use, especially family encouragement, remained significant indicators of digital divides in old age. Gray digital divides are not only, or even primarily, determined by the individual process of physiological aging but are also defined by personal and positional categories within wider society (Sala et al., 2020).

### Typologies of older mobile phone users

Mobile phone usage in later life also has clear patterns and characteristics that have allowed scholars to create typologies of older mobile users. Nimrod (2016) provided a pyramid-like typology of the incorporation of mobile phones in later life, starting from the baseline of voice calls and moving upward via basic functions (i.e., short message service [SMS], camera, and more) and Internet-based functions (social networking sites [SNS], e-mail, and more) before reaching the top, characterized by media players (music, podcasts, and more). Vicente and Lopes (2016) provided a typology that differentiates between “busy and active,” “social and hedonic,” and “apathetic” users. The first category included older people active in the labor market with developed digital skills. The second category included older adults who perceived the mobile phone as a social status object and used a relatively low variety of functionalities. The third category included older users who valued the mobile phone only for remaining in contact with others and who were indifferent to its other functionalities.

Typologies based on cross-sectional research designs may create a false conception of “fixed user categories.” As people get older and experience various life transitions, the ways in which they use mobile phones can undergo changes. Thus, a user's position in these typologies may change over time for several reasons. Older mobile phone users may switch from basic-feature phones to smartphones or the other way around (e.g., Jacobson et al., 2017). Older people may also improve their digital skills, which allows for a broader scope of mobile phone use. For example, they can learn how to send photos or audio files to other users, leading to the augmentation of their digital repertoire (Hänninen et al., 2021) from basic to more advanced communication skills. Third, in accordance with the notion of utilitarian use (Ivan & Fernández-Ardèvol, 2017), some mobile phone functionalities may become irrelevant or less necessary with age, while others may become more useful. For example, following retirement, there may be less need for an alarm clock function. In contrast, following a relative's demise, there may be a greater need for messaging apps to provide support to the family.

### Explaining cross-country differences

In relation to older media users, the lack of longitudinal data collected from multiple countries is well acknowledged. Some rare studies are exceptions to this rule (e.g., Nimrod, 2019; Taipale et al., 2021). In these studies, the detected cross-country differences in older adults’ media use were considerably large, and the effect sizes of country of residence turned out to be larger than those of basic sociodemographic factors (Taipale et al., 2021). The scarcity of this type of study is due not only to the high cost of longitudinal cross-country surveys, but severe challenges also relate to questionnaire localization and to the interpretation of country differences, which are often unsystematic.

To better understand the cross-country differences in older adults’ (mobile) media use changes, at least three perspectives can be outlined. First, the media history in each country provides a context for the integration of different media formats into people's lives (Bolin, 2016). For example, in Western countries, smartphones began to be used around 2007. It took them about 10 years to become staples of people's lives. In the less advanced economies, smartphones were introduced approximately four to five years later (Luty, 2021).

Second, media transitions are not only outcomes of individual preferences and free choice but are shaped by what they can afford in a certain moment of their lives. Recently, attempts have been made to integrate the role of available communication services and pricing schemes into the studied analysis of the types of mobile phone functions that people use in different countries (Fernández-Ardèvol, 2010, 2019). According to this research, North American countries are characterized by higher pricing systems than, for example, many European Union (EU) member states. However, even at the EU level, the pricing system of 4G or 5G subscriptions differs remarkably from country to country. For example, the Netherlands and Finland have the lowest weighted average monthly prices for mobile communication (REWHEEL, 2020). Lower mobile subscription costs, coupled with decent income (e.g., Brown et al., 2011; Taipale, 2016), are most likely contributing to more versatile mobile phone use.

Third, country differences regarding the availability of technology and social support for older adults shape the adoption and use of mobile phones. In particular, family structure, housing arrangements, and geographical distances influence the frequencies and patterns of technological support available to and provided for older family members (Taipale, 2019). The living arrangements of older persons are markedly different in northern Europe (following the aging-in-place policy) compared to the southern and eastern parts of Europe (family care and community care model). For example, the highest percentage of older people (aged 65 or above) living alone are found in Finland, Estonia, and the Netherlands, whereas Mediterranean countries, Israel, and some of the Eastern European countries (Romania and Poland) have the highest percentages of older people living with their families (United Nations, 2019).

## Research questions

The objective of this study is to understand the changes in older people's smartphone use between 2018 and 2020. This observation period started before the outbreak of the COVID-19 pandemic and ended at that time. To this end, the following research questions (RQs) are answered:

RQ1: What are the mobile phone use profiles of older adults?
RQ2: What are the changes in older adults’ mobile phone use profiles within two years of follow-up?
RQ3: What are the cross-country differences in the prevalence of user profiles and transitions between them?
RQ4: How are mobile use profiles and transitions between them related to demographic and socioeconomic characteristics?

Regarding RQ1, based on the literature review, it is assumed that mobile phone use profiles are organized in a hierarchical manner (Nimrod, 2016). Concerning RQ2, it is expected that the changes between the identified use profiles are manifold (Anderson, 2005), indicating the patterns of adoption, dropping out, and re-adoption. With regard to RQ3, it is assumed that significant country differences emerge from the data, as in previous studies (Fernández-Ardèvol, 2010, 2019; Taipale et al., 2021). Lastly, regarding RQ4, it is anticipated that younger age, male gender, higher education, higher income, and employment and residence in urban locations are factors dividing the studied population of older adults.

## Methods

### Data and participants

The data were drawn from the second (2018) and third (2020) waves of the Aging + Communication + Technologies (ACT) cross-national longitudinal study involving Internet users aged between 62 and 96 (in 2018) from Austria, Canada, Finland, Israel, the Netherlands, Romania, and Spain (for the data reports, see Loos et al., 2018, 2019). To our knowledge, this is the only available cross-national panel dataset on older media users. The data were collected online, except in Romania, where the survey was conducted via telephone due to the low rate of Internet users among the older population. The respondents who reported participating in the second and third waves of the survey (N = 4,398) were included in the analyses.

### Statistical procedures

Statistical analyses were carried out in three steps. First, descriptive statistics on the prevalence of mobile phone function use were provided. Second, a latent class analysis (LCA) was conducted separately for both waves to form profiles of mobile phone functions use.^1^ LCA is a method for identifying unmeasured (i.e., latent) class membership among subjects using categorical and/or continuous observed variables. Third, latent transition analysis (LTA), a longitudinal version of LCA, was applied to the two-wave data to examine the probability of transitions between different classes. The LTA model consists of a measurement model for the latent class variable at each time point and a structural model relating the latent class variables to each other and to covariates. All measurement parameters are typically held invariant across time, although this is not necessary (Muthén, 2007; Muthén & Asparouhov, 2011). In steps two and three, the effects of country, demographic, and socioeconomic factors on class memberships and transitions were estimated using a multinomial logistic regression model built into the LCA and LTA models.

### Measurements

Nineteen binary (1 = yes, 0 = no) statements were presented to the respondents in both survey waves to inquire about their use of mobile phones. These statements addressed the following functions: 1) alarm clock and reminders, 2) calendar, 3) downloading apps, 4) e-mail, 5) games, 6) global positioning system (GPS) and maps, 7) instant messaging, 8) listening to podcasts, 9) listening to radio, 10) multimedia messages (MMS) (sending images or sound), 11) ordinary voice calls, 12) recording video, 13) SMS (sending texts), 14) social network sites, 15) taking photographs, 16) using the phone as music player, 17) visiting websites via apps, 18) visiting websites via browser, and 19) watching TV or video. These questions were asked only of the respondents who reported having a mobile phone available in their households (445 respondents were excluded).

As main independent variables, the following measures were related to the respondents’ demographic and socioeconomic backgrounds: gender (1 = male, 0 = female), age (years), marital status (1 = married, 0 = other), employment status (1 = retired, 0 = other), education (0 = primary, 1 = secondary, 2 = tertiary), income (1 = a lot below country average–5 = a lot above country average), and place of residence (0 = countryside, 1 = town, 2 = city). In addition, the respondents’ countries of residence (Austria, Canada, Finland, Israel, the Netherlands, Romania, and Spain) were included in all analyses (for sample distributions, see Supplementary Table 1).

## Results

### Descriptive findings on the use of mobile phone functions

**Figure 1.**
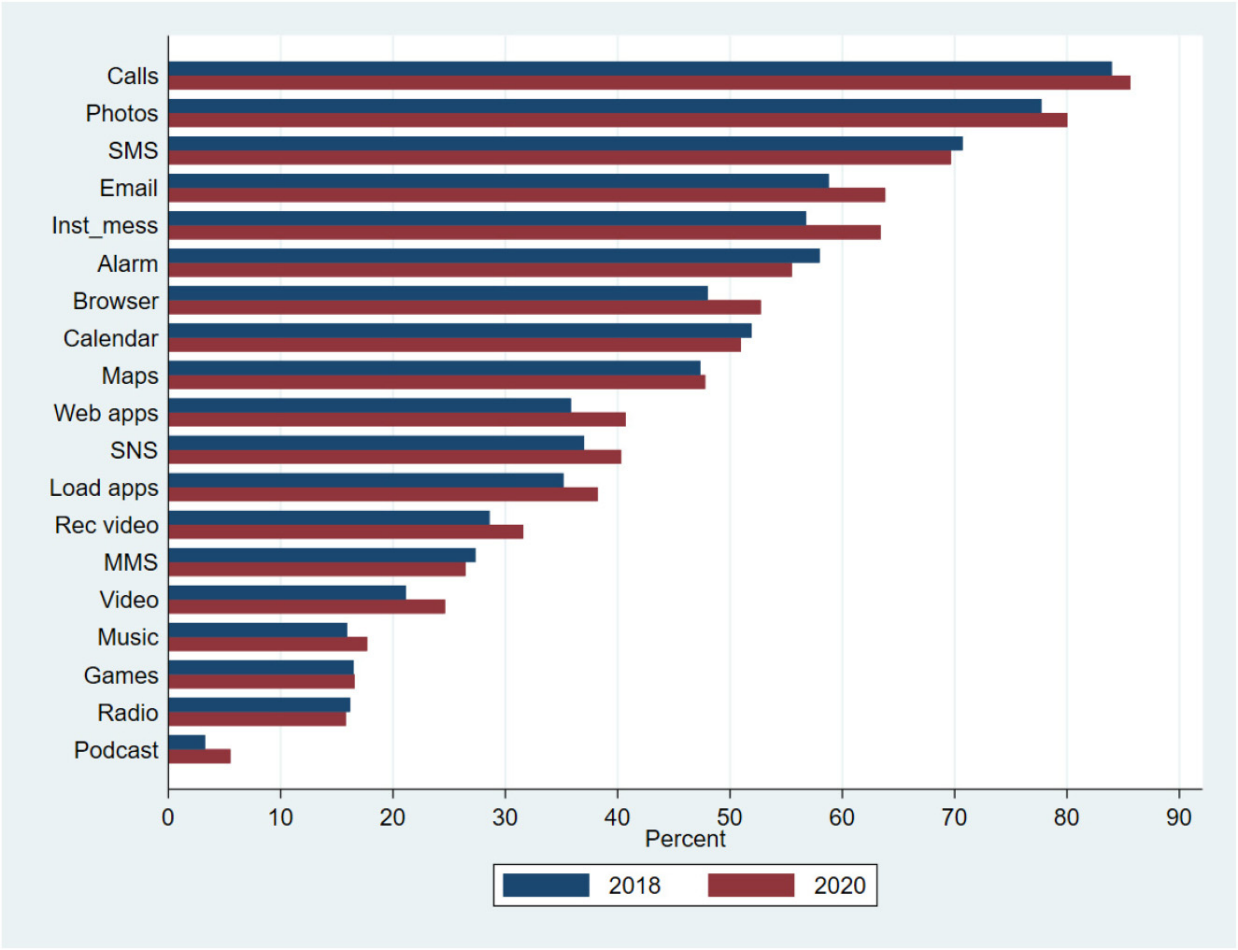
Prevalence of mobile function use in full sample by survey year (2018: N = 7,354; 2020: N = 4,170).

*Note:* Load apps = downloading apps; inst_mess = instant messaging; MMS = multimedia messages; Rec video = recording video; SMS = sending text messages; SNS = social network sites; Web apps = visiting websites via apps; Browser = visiting websites via browser; Video = watching TV or video.

Figure 1 shows the overall prevalence of the use of mobile phone functions by survey year in the full sample, which included respondents from all countries. Older adults’ mobile phone use was predominantly related to social interaction, especially basic functionalities, such as voice calls and SMS. Between 2018 and 2020, the largest increases took place in the use of instant messaging apps, e-mails, and visits to websites via apps or browsers. In contrast, the use of media content, such as listening to podcasts, radio or music, or playing games, remained uncommon during the two-year observation period.

This overall picture masks substantial country differences in the prevalence of specific mobile phone functions and related changes over time (see Supplementary Table 2). Differences in the use of particular mobile functions between the countries were numerous yet, to some extent, systematic. For example, the use of SMS was very common in both waves in all countries except the Netherlands, Romania, and Spain. In Israel and Spain, the use of instant messengers and e-mails was more common than in other countries in both waves.

### Mobile phone use profiles

In the second step of the analysis, the profiles for different mobile function uses were created using LCA. Tetrachoric correlations were calculated to determine how mobile phone functions were clustered. Correlations revealed that some functions were clearly used together (e.g., taking photos and recording videos) (see Supplementary Table 3) and that they might form clusters of different functions (cf. clustering of individuals). However, an exploratory factor analysis conducted on the tetrachoric correlation matrix clearly favored a unidimensional solution, as did a multiple correspondence analysis with the same items. Thus, even if some mobile phone functions were more closely interrelated than others, the 19 items clearly measured one underlying dimension of mobile phone function use.

**Figure 2.**
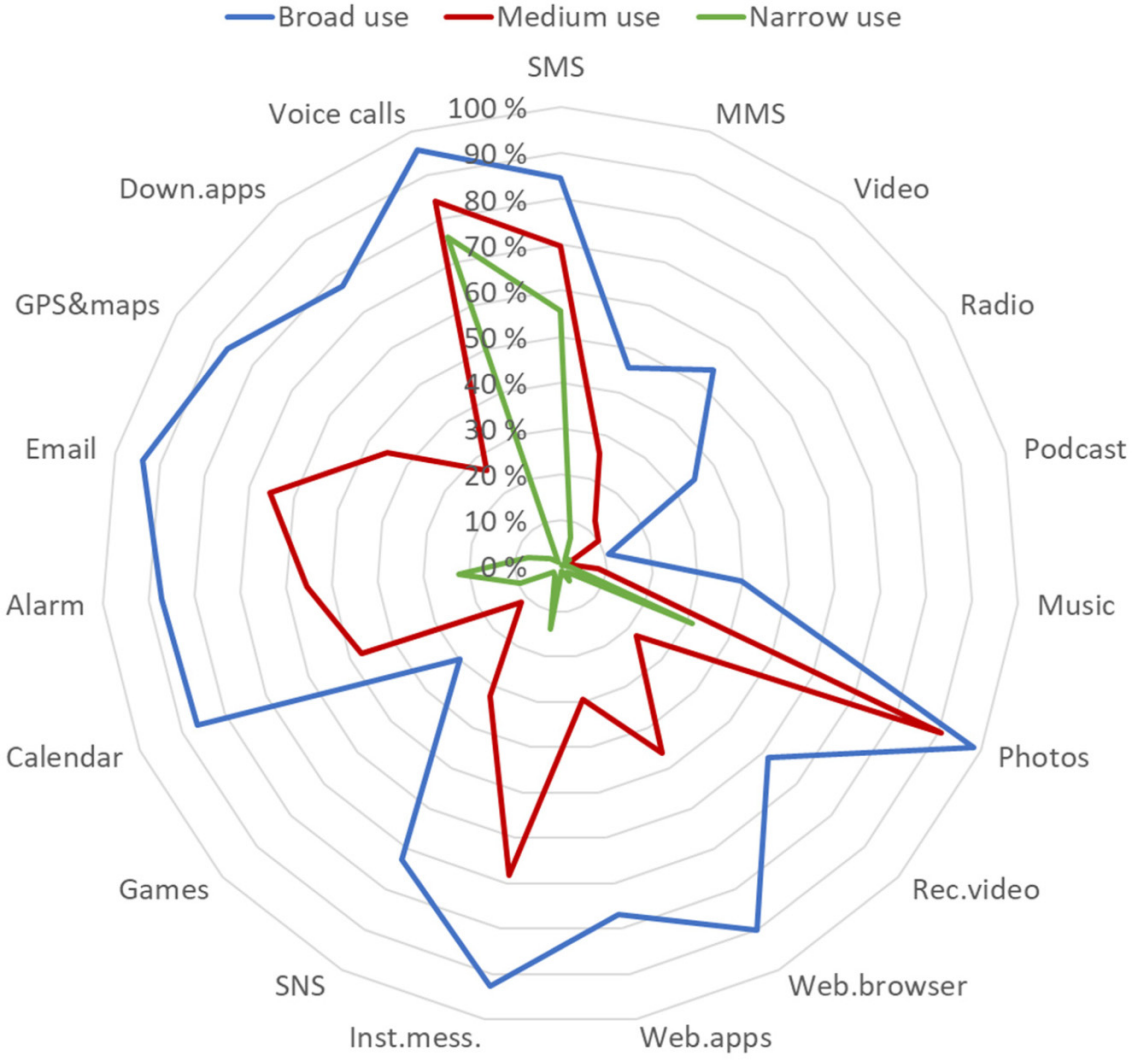
Mobile phone functions use profiles (LCA) in the pooled 2018–2020 data.

*Note:* Down.apps = downloading apps; inst.mess = instant messaging; MMS = multimedia messages; Rec.video = recording video; SMS = sending text messages; SNS = social network sites; Web.apps = visiting websites via apps; Web.browser = visiting websites via browser; Video = watching TV or video.

According to the fit indices (Bayesian information criterion [BIC] and Bayesian information criterion [ABIC]), a three-class model combined good fit and parsimony in both survey waves and in all countries (Supplementary Figures 1 and 2). Figure 2 presents the estimated probabilities of the use of individual functions within each of the three latent classes in the pooled data. The first class (labeled “Broad Use”) was marked by the broad use of various mobile phone functions. In contrast, the third class (labeled “Narrow Use”) was marked by using mobile phones for voice calls and SMS and, to a lesser degree, for taking photos. In terms of the variety of mobile phone functions, the second class (labeled “Medium Use”) fell between the first and third classes.

**Figure 3.**
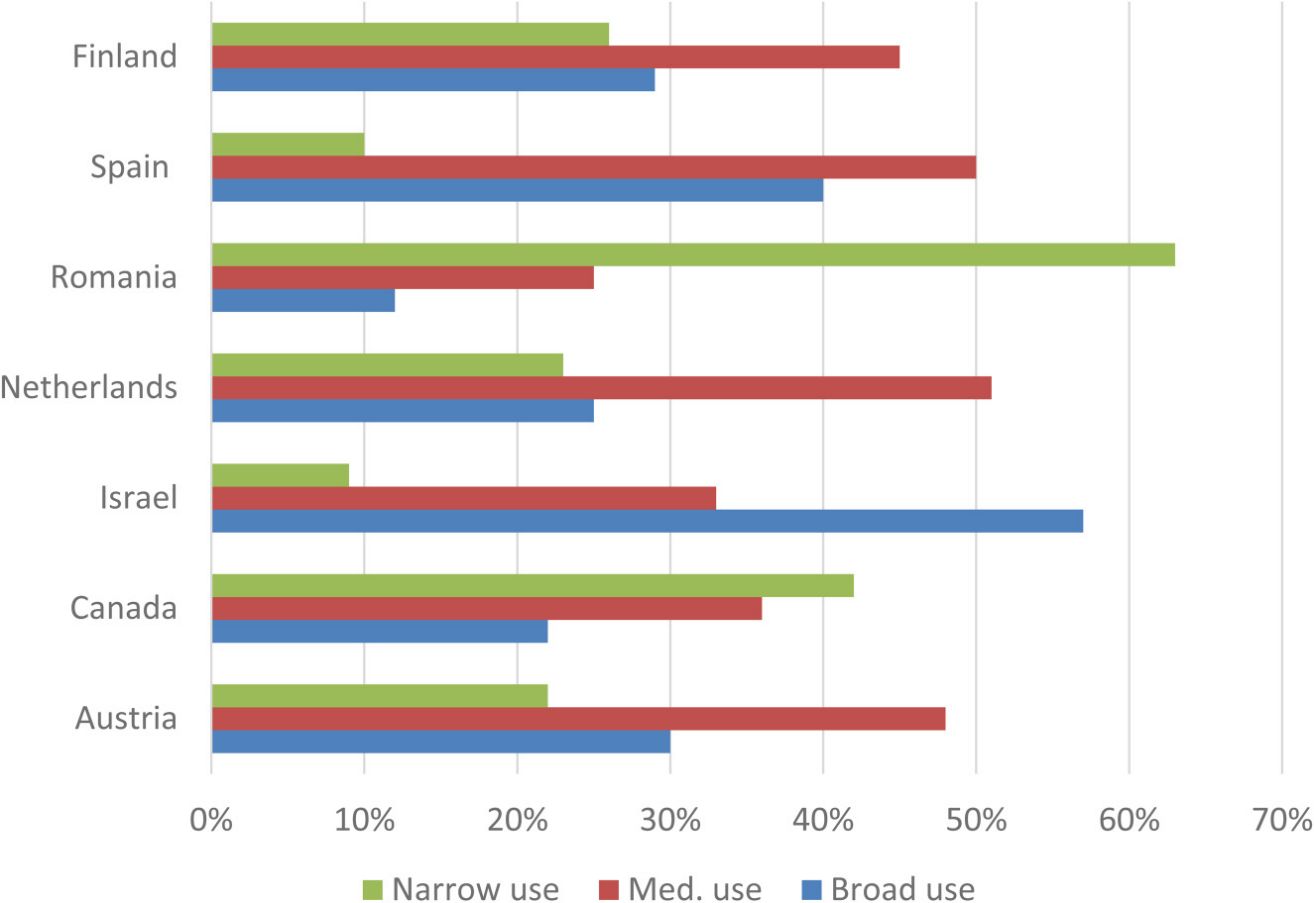
Prevalence of mobile phone use profiles by country in 2018.

In Figure 3, the prevalence of the three profiles in 2018 is presented by country. Considerable differences were found between the seven countries. The share of the “Narrow Use” profile was highest in Romania (63%), while the “Broad Use” profile was the most common in Israel (57%). In the other five countries, the most common class was “Medium Use,” yet regarding the prevalence of “Broad Use,” cross-country differences were notably large. In addition to Israel, the “Broad Use” class was relatively common in Spain, Finland, and Austria.

### Predicting belonging to mobile use profiles

The characteristics of the respondents belonging to the three mobile use profiles were analyzed using a multinomial logistic model built into the LTA framework. According to the results (Table 1), older respondents were less likely to belong to the “Broad Use” or “Medium Use” profiles than to the “Narrow Use” profile, which served as a reference class. Compared to the “Narrow Use” profile, married respondents were more likely to belong to the “Broad Use” profile than unmarried respondents (all others). Education had a clear positive effect on use profiles; the higher the education, the broader the scope of the mobile functions used. Relative income turned out to be a strong predictor of both the “Medium Use” and “Broad Use” profiles. Respondents with higher incomes were more likely to use a wider array of mobile phone functions than those with lower incomes. A statistically significant effect was also found for the respondents living in cities, who were more likely to belong to the “Broad Use” profile than the respondents living in the countryside or village.

**Table 1.** Multinomial logistic model predicting belonging to different mobile function use profiles (LCA) in 2018 (N = 3,546)^a^.

	Medium Use	Broad Use
Female (ref.)	1	1
Male	0.82°	1.14
Age	0.94***	0.89***
Other (ref.)	1	1
Married	1.23	1.40*
Other (ref.)	1	1
Retired	1.05	0.91
Primary education (ref.)	1	1
Secondary education	1.42	2.00*
Tertiary education	1.78*	3.02**
Income lot below average (ref.)	1	1
Income below average	1.17	1.38
Income average	1.48*	1.35
Income above average	1.74**	2.03**
Income a lot above average	2.13*	3.54***
Countryside & village (ref.)	1	1
Town & Suburb	1.01	1.19
City	1.25	1.38*

a Reference class: Narrow Use. Coefficients are odds ratios. Controlling for country.

°*p* < 0.10; *p < 0.05; **p < 0.01; ***p < 0.001

### Changes in mobile phone use profiles by country

**Figure 4.**
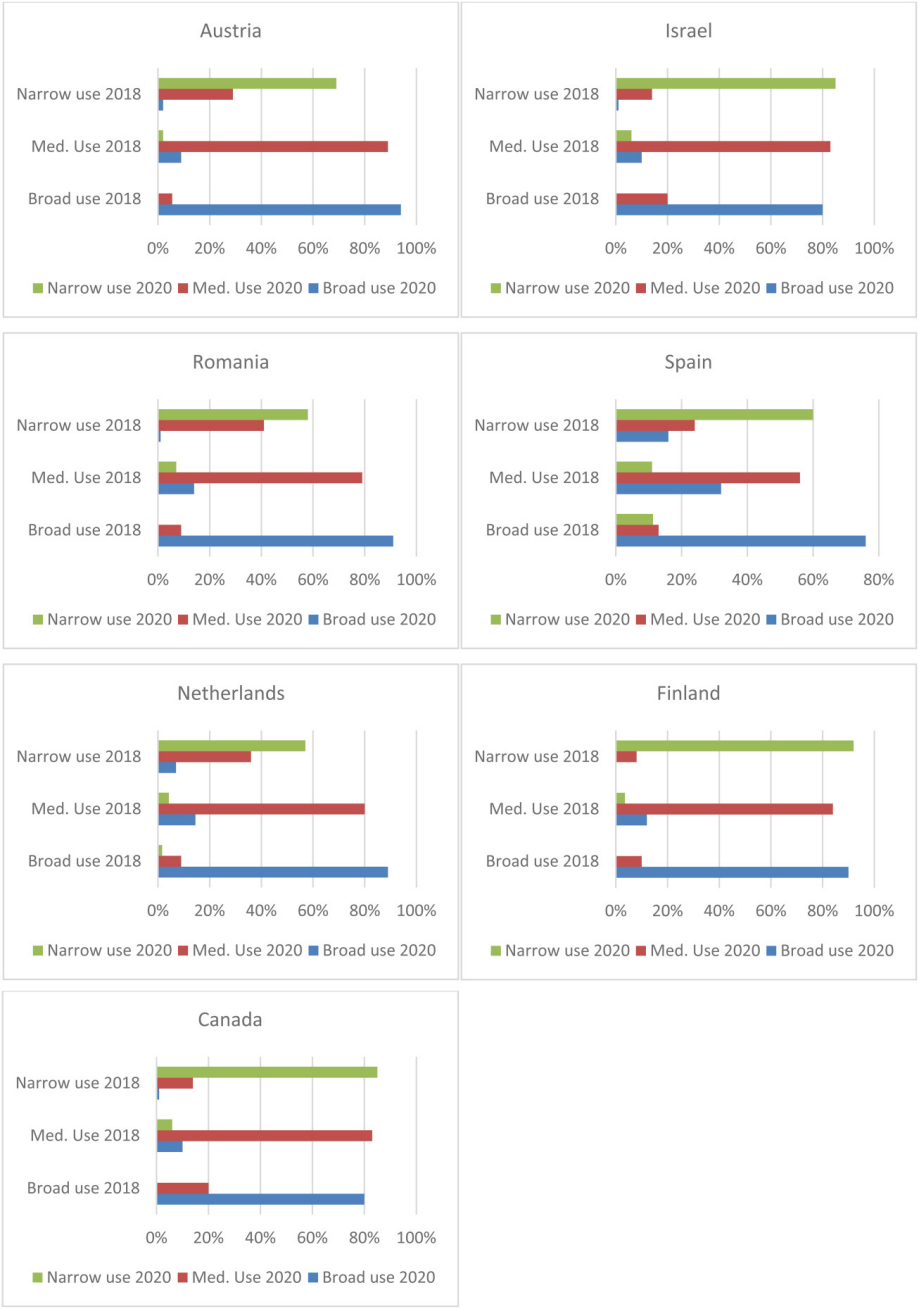
Changes in mobile function use profiles by country between 2018 (y-axis) and 2020 (legend).

The transition probabilities between the three profiles in all seven countries were studied next, and large cross-country differences, similar to previous analyses, were discovered (Figure 4). Transitions between classes were most common in Romania. In contrast, the transitions were uncommon (i.e., high-class stability) in other countries, especially Finland, Israel, and Canada. In Austria, the Netherlands, and Romania, the “Narrow Use” profile had the lowest stability. This means that transitions typically took place from the “Narrow Use” profile toward the “Medium Use” and “Broad Use” profiles. In contrast, stability was highest in the “Broad Use” profile in most of the countries. Taken together, these findings indicate that the respondents were more likely to broaden than narrow the scope of mobile phone functions as they became older.

### Predicting the likelihood of mobile use profile changes by baseline characteristics^2^

The differences in transition probabilities regarding the respondents’ demographic and socioeconomic characteristics at the beginning of the observation period were examined using a multinomial logistic model (Table 2). Since transitions between mobile phone use profiles were rare (see Figure 4), the estimation of some effects failed because there were not enough cases. In particular, the estimation of the effect of education, income, and place of residence was not possible for the respondents who belonged to the “Broad Use” profile in 2018.

**Table 2.** Multinomial logistic model on the effect of life situation (in 2018) on changes in mobile phone function profiles between 2018 and 2020.

	Profile in 2018					
	Broad Use		Medium Use		Narrow Use	
	Profile in 2020^a^					
	Medium Use	Broad Use	Medium Use	Broad Use	Medium Use	Broad Use
Female (ref.)	1	1	1	1	1	1
Male	1.95	2.71	0.48**	0.41***	0.96	1.15
Age	0.88	0.86	1.01	0.93°	0.96*	0.85**
Other (ref.)	1	1	1	1	1	1
Married	0.49	0.27*	2.29	2.26	0.69*	1.28
Other (ref.)	1	1	1	1	1	1
Retired	2.16	2.18	1.07	1.18	1.23	1.57
Primary education (ref.)	1	1	1	1	1	1
Secondary education	—	—	0.82	0.94	0.74	—
Tertiary education	—	—	1.06	1.70	0.92	—
Income below average (ref.)	1	1	1	1	1	1
Income average	—	—	1.39	2.32	1.14	0.79
Income above average	1.24	1.79	1.51	2.23	1.29	0.62
Countryside & village (ref.)	1	1	1	1	1	1
Town & suburb	—	—	0.53*	0.85	0.72	1.37
City	—	—	0.62	0.92	0.88	1.58

a Reference class is Narrow Use. Coefficients are odds ratios. Controlling for country.

°*p* < 0.10; *p < 0.05; **p < 0.01; ***p < 0.001

The results show that being married decreased the probability of staying in the “Broad Use” profile. Among the respondents who belonged to the “Medium Use” profile in 2018, being male decreased the probability of switching to the “Broad Use” profile. Men also had a lower probability of staying in the “Medium Use” profile than women. Similarly, place of residence was a significant predictor of staying in the “Medium Use” profile, although the large effect size should be interpreted with caution. Again, living in a town or a suburb decreased the probability of switching to the “Medium Use” profile compared to staying in the “Narrow Use” profile. Regarding those who belonged to the “Narrow Use” profile in 2018, it was found that higher age lowered the probability of transition to the “Broad Use” or “Medium Use” profiles. Overall, the effects of demographic and socioeconomic characteristics on transition probabilities were relatively scarce.

## Discussion and conclusions

The overall aim of this study was to investigate changes in older adults’ mobile phone use from the pre-COVID-19 (2018) to the COVID-19 (2020) period. The analysis began with bivariate methods, which revealed possible functional displacements between individual mobile phone functions. Among the respondents, the use of instant messaging increased significantly over the two-year period, while functionalities related to social interactions, such as calls and SMS, were predominantly utilized. Notably, there were no substantial declines in the use of any specific mobile phone function. Thus, the recorded increase in instant messaging aligns with the augmentation thesis (e.g., Shklovski et al., 2004), suggesting that the adoption of new functionalities expands the scope of mobile phone usage without replacing existing ones.

Regarding RQ1, dealing with mobile phone user profiles, the study provided a clear and statistically robust outcome; the three mobile use profiles, which differ from each other in terms of the breadth of the mobile phone functions used, were identified from the data. This three-profile model aligns well with Nimrod's (2016) pyramid-like typology of mobile phone use in later life. Respondents in the “Narrow Use” profile mainly used older basic functionalities—voice call and SMS—and the scope of function broadened in a cumulative way toward the “Broad Use” profile.

RQ2 dealt with the changes in older adults’ mobile phone use profiles from before to during the COVID-19 pandemic. The findings showed high stability in mobile phone function use over time, and the identified transitions between the profiles were rare. The changes took place mainly from “Narrow Use” to “Medium Use” and “Broad Use.” This outcome follows the logic of augmentation thesis (Shklovski et al., 2004): older adults are more likely to expand their mobile phone use than to slide back to the use of basic functions only. In addition, high stability in the “Broad Use” profile deserves attention. This suggests that older adults are loyal users of mobile phones (for media loyalty, see Nimrod, 2019), who do not easily abandon communication channels and other mobile functions once they have adopted them. Considering that Gallistl and Nimrod (2020) showed a decrease in the time spent on different media formats among older adults in just two years’ time span, the scarcity of transitions and high stability in certain profiles are somewhat unexpected results.

RQ3 asked about cross-country differences concerning the prevalence of user profiles and transitions between them. The cross-sectional analysis revealed a clear digital divide between Romania and the other six countries in 2018 regarding the “Narrow Use” and “Broad Use” profiles. Romanian respondents had not yet adopted more advanced functionalities in 2018, whereas a relatively broad array of mobile functions was already used in Israel and, to a lesser extent, in Spain, Finland, and Austria. Regarding the transitions between the three profiles, country differences were also notably large. The transitions from the “Narrow Use” profile toward a broader scope of use were the most common in Romania. In countries where “Medium Use” and “Broad Use” profiles were more prevalent in 2018, such as Israel and Finland, the stability of each profile was high. Based on these findings, it may be concluded that gaps between countries in older adults’ mobile phone use are perhaps slowly narrowing.

The detected country differences in prevalence and transitions can also be discussed in connection with the three interpretive frameworks presented in the literature review. Regarding media history, it is likely that Israeli, Spanish, Finnish, and Austrian respondents had longer personal mobile phone use trajectories compared with Romanian respondents. Therefore, starting with a more limited scope of mobile phone functions in 2018, Romanian respondents were able to catch up with others in a relatively short period of time. Second, income turned out to be a strong predictor, suggesting that lower levels of income in Romania may have slowed down the transition toward more extensive use of mobile phones. Third, due to the more limited availability of public services, support, and training in digital skills in Romania, older adults may have been more dependent on family and friends while learning to use new features of their mobile phones.

Lastly, RQ4 was defined to clarify the ways in which the mobile phone use profiles and transitions between them were related to demographic and socioeconomic characteristics. Cross-sectional analysis showed that older age (in 2018) decreased the likelihood of using a broader range of mobile functions, yet income differences were more determinative in this respect. Older adults with income a lot above national average were 3.5 times likelier to belong to the “Broad Use” category than to the “Narrow Use” category. Compared to other statistically significant factors, marital status and place of residence had only marginal effects. Based on all this, it can be concluded that the gray digital divide in mobile phone use is intertwined, especially with income differences.

Regarding the transitions, it was found that the older the respondents were in 2018, the less likely they were to transition from “Narrow Use” to the other two profiles. In fact, of all the studied demographic and socioeconomic characteristics, the effect of age was most systematically related to the transitions between the three profiles. This finding substantiates the idea that “progressive transitions” in mobile phone use become less likely as older people become older. In other words, the gray digital divide in mobile phone use is less prone to shrink over time among the oldest. This result challenges the aging-and-innovation discourse (see Neven & Peine, 2017), which presents a neoliberal view of aging that normalizes new and more innovative technologies as acceptable solutions to later-life “problems.” In contrast, the findings are better aligned with studies (e.g., Caliandro et al., 2021b) that demonstrate that the COVID-19 pandemic did not result in dramatic changes in the social media practices of older adults. Except for age, the transitions between the mobile function use profile were marginally predicted by male gender, being married, and living in towns or suburbs in 2018. However, compared to age, these associations were unsystematic, indicating that they were not constitutive of any clear-cut and distinctive digital divides in later life.

To summarize, the present study demonstrated that the so-called gray divide in mobile phone use is not yet close to being bridged (see Friemel, 2016 for Internet use). The breadth of mobile use is also strongly dependent on age among the oldest Internet users. A longitudinal panel design provided added value compared to earlier research. This made it possible to show that the “progressive” transitions toward a wider spectrum of mobile phone function use become less likely with age. The finding seems to be in line with prior research, suggesting that after the age of 75, media users have increasingly difficulty adapting and adjusting to new forms of media (Loos & Ivan, 2022). In this respect, the gray divide in mobile use appears to be a relatively persistent phenomenon. However, the study also disclosed some positive aspects of mobile phone use in later life. Among the studied population, it was uncommon for the scope of mobile phone use to have substantially narrowed (i.e., transitions from “Broad Use” to “Narrow Use”) during the two-year observation period. Instead, the respondents either continued to use the same functionalities or adopted new ones. Hence, this study provides considerably more support for the augmentation thesis than for functional displacement or time displacement theses. Moreover, and against what the public discourse may suggest, it seems that the COVID-19 pandemic did not, at least markedly, widen the spectrum of mobile phone use among the older Internet user population. Additionally, the progressive steps in mobile phone use are typically small.

## Limitations and future research

The study has some limitations that should be considered. First, the target population consisted of relatively highly educated older adults who were Internet users and, hence, probably proficient in smartphone use, which limits the generalizability of the findings to the overall population of the same age group. Second, the follow-up period of two years is relatively short for significant changes in mobile phone use to occur, particularly considering that older adults tend to be loyal users of established media formats. More time may be needed for them to adapt to new media or communication channels. Additionally, the dataset had limited representation of substantial life changes, such as retirement, changes in marital status, income, and place of residence. Apart from these two major limitations, it is worth noting that the dataset did not include some potentially important predictors. For example, indicators of the respondents’ personal media histories and the availability of digital support were not available. Likewise, many cultural factors (e.g., privacy concerns and favored communication modalities) affecting the use of digital technologies were not measured. Future research should address these limitations by examining the connections between old-age life transitions and media transitions over a longer observation period. This may reveal stronger associations between specific mobile functions and life transitions. For example, changes in e-mail use may be linked to retirement transitions, while the adoption of social media and instant messaging could be influenced by changes in marital status or grandparenthood in later life.

## Supplemental Material

sj-docx-1-mmc-10.1177_20501579231185479 - Supplemental material for Mobile phone use before and during the COVID-19 pandemic – a panel study of older adults in seven countries Click here for additional data file. Supplemental material, sj-docx-1-mmc-10.1177_20501579231185479 for Mobile phone use before and during the COVID-19 pandemic – a panel study of older adults in seven countries by Sakari Taipale, Tomi Oinas, Loredana Ivan and Dennis Rosenberg in Mobile Media & Communication

## Notes

1. LCA is a mixture model, which means that latent variables are categorical, unlike in structural equation modeling and factor analysis models (EFA and CFA), which are based on continuous latent variables. In contrast to clustering methods, LCA considers that there is a measurement error in assigning people to different classes.

2. The effects of life transitions (becoming retired, changes in urbanity of residence, changes in education and income) between 2018 and 2020 on changes in mobile phone use profiles were also studied. However, it turned out that a small number of life transitions coupled with a scarcity of media transitions considerably lowered the statistical power, and no reliable solution was reached.

## References

[bibr1-20501579231185479] Anderson B. (2005). The value of mixed-method longitudinal panel studies in ICT research: Transitions in and out of ‘ICT poverty’ as a case in point. Information, Community & Society*,* 8(3), 343–367. 10.1080/13691180500259160

[bibr2-20501579231185479] Bolin G. (2016). Media generations: Experience, identity and mediatised social change*.* Routledge. 10.4324/9781315694955

[bibr3-20501579231185479] Brown K. Campbell S. W. Ling R. (2011). Mobile phones bridging the digital divide for teens in the US? Future Internet*,* 3(2), 144–158. 10.3390/fi3020144

[bibr4-20501579231185479] Caliandro A. Garavaglia E. Anselmi G. (2021a). Studying ageism on social media. An exploration of ageing discourses related to COVID-19 in the Italian twittersphere. Rassegna Italiana di Sociologia, 62(2), 343–375. 10.1423/101848

[bibr5-20501579231185479] Caliandro A. Garavaglia E. Sturiale V. Di Leva A. (2021b). Older people and smartphone practices in everyday life: An inquire on digital sociality of Italian older users. The Communication Review, 24(1), 47–78. 10.1080/10714421.2021.1904771

[bibr6-20501579231185479] Casanova G. Abbondanza S. Rolandi E. Vaccaro R. Pettinato L. Colombo M. Guaita A. (2021). New older users’ attitudes toward social networking sites and loneliness: The case of the oldest-old residents in a small Italian city. Social Media + Society*,* 7(4), 10.1177/20563051211052905

[bibr7-20501579231185479] Cavapozzi D. Dal Bianco C. (2022). Does retirement reduce familiarity with information and communication technology? Review of Economics of the Household, 20(2), 553–577. 10.1007/s11150-021-09573-8 34248449 PMC8254456

[bibr8-20501579231185479] De Waal E. Schoenbach K. (2010). News sites’ position in the mediascape: Uses, evaluations and media displacement effects over time. New Media & Society, 12(3), 477–499. 10.1177/1461444809341859

[bibr9-20501579231185479] Fernández-Ardèvol M. (2010). Interactions with and through mobile phones: What about the elderly population? 3rd European Communication Conference (ECREA), Hamburg, Germany. http://hdl.handle.net/10609/7001

[bibr10-20501579231185479] Fernández-Ardèvol M. (2019). One phone, two phones, four phones: Older women and mobile telephony in Lima, Peru. In C. Wamala Larsson & L. Stark (Eds.), Gendered power and mobile technology (pp. 93–107). Routledge.

[bibr11-20501579231185479] Fernández-Ardèvol M. Ivan L. (2013). Older people and mobile communication in two European contexts. Romanian Journal of Communication & Public Relations*,* 15(3), 83–98.

[bibr12-20501579231185479] Fernández-Ardèvol M. Sawchuk K. Grenier L. (2017). Maintaining connections: Octo-and nonagenarians on digital ‘use and non-use’. Nordicom Review, 38(s1), 39–51. 10.1515/nor-2017-0396

[bibr13-20501579231185479] Fortunati L. Bakardjieva M. (2020). Mobile Convergence. In R. Ling, L. Fortunati, G. Goggin, S. S. Lim, & Y. Li (Eds.), The Oxford handbook of mobile communication and society (pp. 81–92). Oxford University Press.

[bibr14-20501579231185479] Fortunati L. Taipale S. (2014). The advanced use of mobile phones in five European countries. The British Journal of Sociology*,* 65(2), 317–337. 10.1111/1468-4446.12075 24697752

[bibr15-20501579231185479] Friemel T. N. (2016). The digital divide has grown old: Determinants of a digital divide among seniors. New Media & Society*,* 18(2), 313–331. 10.1177/1461444814538648

[bibr16-20501579231185479] Gallistl V. Nimrod G. (2020). Media-based leisure and wellbeing: A study of older internet users. Leisure Studies, 39(2), 251–265. 10.1080/02614367.2019.1694568

[bibr17-20501579231185479] Hänninen R. Taipale S. Luostari R. (2021). Exploring heterogeneous ICT use among older adults – the warm experts’ perspective. New Media and Society*,* 23(6), 1584–1601. 10.1177/1461444820917353

[bibr18-20501579231185479] Humphreys L. Von Pape T. Karnowski V. (2013). Evolving mobile media: Uses and conceptualizations of the mobile internet. Journal of Computer-Mediated Communication*,* 18(4), 491–507. 10.1111/jcc4.12019

[bibr19-20501579231185479] Ivan L. Fernández-Ardèvol M. (2017). Older people and the use of ICTs to communicate with children and grandchildren. Transnational Social Review*,* 7(1), 41–55, 10.1080/21931674.2016.1277861

[bibr20-20501579231185479] Jacobson J. Lin C. Z. McEwen R. (2017). Aging with technology: Seniors and mobile connections. Canadian Journal of Communication*,* 42(2), 331–357. 10.22230/cjc.2017v42n2a3221

[bibr21-20501579231185479] Jenkins H. (2006). Convergence culture: Where old and new media collide. New York University Press.

[bibr22-20501579231185479] Kayany J. M. Yelsma P. (2000). Displacement effects of online media in the socio-technical contexts of households. Journal of Broadcasting & Electronic Media, 44(2), 215–229. 10.1207/s15506878jobem4402_4

[bibr23-20501579231185479] Kim S. J. Viswanathan V. Lee H. M. (2020). Platform war vs. Platform synergy? A longitudinal analysis of media substitution between personal computers and mobile devices. Journal of Broadcasting & Electronic Media, 64(2), 65–88. 10.1080/08838151.2020.1718986

[bibr24-20501579231185479] Lee S. Y. Lee S. W. (2015). Online video services and other media: Substitutes or complement. Computers in Human Behavior, 51(Part A), 293–299. 10.1016/j.chb.2015.03.073

[bibr25-20501579231185479] Loos E. Ivan L. (2022). Not only people are getting old, the new media are too: Technology generations and the changes in new media use. New Media & Society, 0(0). 10.1177/14614448221101783

[bibr26-20501579231185479] Loos E. F. Nimrod G. Fernández-Ardèvol M. (Eds.). (2018). Older audiences in the digital media environment: A cross-national longitudinal study. Wave 1 Report v1.0. ACT Project, Concordia University. https://spectrum.library.concordia.ca/id/eprint/983866/1/Loos_Nimrod_FernandezArdevol_2018_final.pdf

[bibr27-20501579231185479] Loos E. F. Nimrod G. Fernández-Ardèvol M. (Eds.). (2019). Older audiences in the digital media environment: A cross-national longitudinal study. Wave 2 Report v2.0. ACT Project, Concordia University. https://spectrum.library.concordia.ca/id/eprint/986444/1/Loss_nimrod_Fernandez_Longitunidal_wv2_2019.pdf

[bibr28-20501579231185479] Luty J. (2021). Smartphone penetration rate in CEE region 2021, by country*.* https://statista.com/statistics/1134059/smartphone-reach-in-cee-region

[bibr29-20501579231185479] Marston H. R. Musselwhite C. Hadley R. (2020, March 18). COVID-19 vs social isolation: The impact technology can have on communities, social connections and citizens. Aging Issues. https://ageingissues.wordpress.com/2020/03/18/covid-19-vs-social-isolation-the-impact-technology-can-have-on-communities-social-connections-and-citizens/

[bibr30-20501579231185479] McCombs M. (1972). Mass media in the marketplace. Journalism Monographs*,* 24, 1–104.

[bibr31-20501579231185479] Melis G. Sala E. Zaccaria D. (2022). The role of information and communication technologies in researching older people during the COVID-19 pandemic. In Q. Gao & J. Zhou (Eds.), Human aspects of IT for the aged population (pp. 53–68). Springer. 10.1007/978-3-031-05581-2_5

[bibr32-20501579231185479] Millward P. (2003). The ‘grey digital divide’: Perception, exclusion and barriers of access to the Internet for older people. First Monday, 8(7). 10.5210/fm.v8i7.1066

[bibr33-20501579231185479] Muthén B. (2007) Latent variable hybrids: Overview of old and new models. In G. R. Hancock & K. M. Samuelsen (Eds.), Advances in latent variable mixture models (pp. 1–24). Information Age Publishing Inc.

[bibr34-20501579231185479] Muthén B. Asparouhov T. (2011). LTA in Mplus: Transition probabilities influenced by covariates. Mplus Web Notes: No. 13. July 27, 2011. https://www.statmodel.com/examples/LTAwebnote.pdf

[bibr35-20501579231185479] Neven L. Peine A. (2017). From triple win to triple sin: How a problematic future discourse is shaping the way people age with technology. Societies, 7(3), 26. 10.3390/soc7030026

[bibr36-20501579231185479] Newell J. Pilotta J. J. Thomas J. C. (2008). Mass media displacement and saturation. The International Journal on Media Management, 10(4), 131–138. 10.1080/14241270802426600

[bibr37-20501579231185479] Nimrod G. (2016). The hierarchy of mobile phone incorporation among older users. Mobile Media & Communication*,* 4(2), 149–168. 10.1177/2050157915617336

[bibr38-20501579231185479] Nimrod G. (2019). Selective motion: Media displacement among older internet users. Information, Communication & Society*,* 22(9), 1269–1280. 10.1080/1369118X.2017.1414865

[bibr39-20501579231185479] Norris P. (2001). Digital divide: Civic engagement, information poverty, and the internet worldwide*.* Cambridge University Press. 10.1017/CBO9781139164887

[bibr40-20501579231185479] Official Statistics of Finland (OSF) (2022). Use of information and communications technology by individuals. Statistics Finland. http://www.stat.fi/til/sutivi/tau_en.html

[bibr41-20501579231185479] Petrovčič A. Taipale S. Rogelj A. Dolničar V. (2018). Design of mobile phones for older adults: An empirical analysis of design guidelines and checklists for feature phones and smartphones. International Journal of Human–Computer Interaction, 34(3), 251–264. 10.1080/10447318.2017.1345142

[bibr42-20501579231185479] REWHEEL. (2020). Rewheel Research/ 4G&5G connectivity competitiveness 2020*.* https://research.rewheel.fi/downloads/4G_5G_connectivity_competitiveness_2020_PUBLIC_VERSION.pdf

[bibr43-20501579231185479] Rolandi E. Vaccaro R. Abbondanza S. Casanova G. Pettinato L. Colombo M. Guaita A. (2020). Loneliness and social engagement in older adults based in lombardy during the COVID-19 lockdown: The long-term effects of a course on social networking sites use. International Journal of Environmental Research and Public Health*,* 17(21), 7912. 10.3390/ijerph17217912 33126634 PMC7662584

[bibr44-20501579231185479] Sala E. Gaia A. Cerati G. (2020). The gray digital divide in social networking site use in Europe: Results from a quantitative study. Social Science Computer Review*,* 40(2), 328–345. 10.1177/0894439320909507

[bibr45-20501579231185479] Seifert A. (2020). The digital exclusion of older adults during the COVID-19 pandemic. Journal of Gerontological Social Work, 63(6–7), 674–676. 10.1080/01634372.2020.1764687 32401181

[bibr46-20501579231185479] Seifert A. Schelling H. R. (2015). Mobile use of the internet using smartphones or tablets by Swiss people over 65 years. Gerontechnology*,* 14(1), 57–62. 10.4017/gt.2015.14.1.006.00

[bibr47-20501579231185479] Shklovski I. Kraut R. Rainie L. (2004). The internet and social participation: Contrasting cross-sectional and longitudinal analyses. Journal of Computer-Mediated Communication*,* 10(1), JCMC1018. 10.1111/j.1083-6101.2004.tb00226.x

[bibr48-20501579231185479] Taipale S. (2013). The relationship between internet use, online and printed newspaper reading in Finland: Investigating the direct and moderating effects of gender. European Journal of Communication*,* 28(1), 5–18, 10.1177/0267323112453672

[bibr49-20501579231185479] Taipale S. (2016). Do the mobile-rich get richer? Internet use, travelling and social differentiations in Finland. New Media & Society*,* 18(1), 44–61. 10.1177/1461444814536574

[bibr50-20501579231185479] Taipale S. (2019). Intergenerational connections in digital families. Springer.

[bibr51-20501579231185479] Taipale S. Oinas T. Karhinen J. (2021). Heterogeneity of traditional and digital media use among older adults: A six-country comparison. Technology in Society*,* 66, 101642. 10.1016/j.techsoc.2021.101642

[bibr52-20501579231185479] Tsatsou P. (2011). Digital divides revisited: What is new about divides and their research? Media, Culture & Society*,* 33(2), 317–331. http://mcs.sagepub.com/cgi/doi/10.1177/0163443710393865

[bibr53-20501579231185479] United Nations. (2019). Population facts. Living arrangements of older persons around the world*.* www.un.org/en/development/desa/population/publications/pdf/popfacts/PopFacts_2019-2.pdf

[bibr54-20501579231185479] van Dijk J. A. G. M. (2005). The deepening divide: Inequality in the information society*.* Sage.

[bibr55-20501579231185479] Vicente P. Lopes I. (2016). Attitudes of older mobile phone users towards mobile phones. Communications*,* 41(1), 71–86. 10.1515/commun-2015-0026

[bibr56-20501579231185479] Wang D. Xiang Z. Fesenmaier D. R. (2016). Smartphone use in everyday life and travel. Journal of Travel Research*,* 55(1), 52–63. 10.1177/0047287514535847

[bibr57-20501579231185479] Westlund O. Ghersetti M. (2015). Modelling news media use: Positing and applying the GC/MC model to the analysis of media use in everyday life and crisis situations. Journalism Studies*,* 16(2), 133–151. 10.1080/1461670X.2013.868139

