# Supplementary material for: Mobile phone use before and during the COVID-19 pandemic – a panel study of older adults in seven countries
Source: Mob Media Commun. 2023 Jul 10:20501579231185479. doi: 10.1177/20501579231185479 (PMC10331323; doi:10.1177/20501579231185479)
Supplement: sj-docx-1-mmc-10.1177_20501579231185479 - Supplemental material for Mobile phone use before and during the COVID-19 pandemic – a panel study of older adults in seven countries [file sj-docx-1-mmc-10.1177_20501579231185479.docx]

Supplementary Table 1. Sample distribution of independent variables in 2018

|  | % / mean | n |
| --- | --- | --- |
| Female | 46% | 2,016 |
| Male | 54% | 2,369 |
|  |  |  |
| Age | 68.5 | 4,396 |
|  |  |  |
| Married | 71% | 3,112 |
| Other | 29% | 1,257 |
|  |  |  |
| Retired | 79% | 3,472 |
| Other | 21% | 924 |
|  |  |  |
| Primary education | 8% | 352 |
| Secondary education | 51% | 2,238 |
| Tertiary education | 41% | 1,795 |
|  |  |  |
| Income lot below average | 22% | 869 |
| Income below average | 15% | 590 |
| Income average | 19% | 729 |
| Income above average | 28% | 1,092 |
| Income a lot above average | 16% | 639 |
|  |  |  |
| Countryside & village | 20% | 888 |
| Town & suburb | 31% | 1,359 |
| City | 49% | 2,132 |
|  |  |  |
| Austria | 17% | 746 |
| Canada | 12% | 516 |
| Israel | 11% | 481 |
| Netherlands | 10% | 438 |
| Romania | 11% | 501 |
| Spain | 21% | 943 |
| Finland | 18% | 773 |

Supplementary Table 2. Prevalence of mobile function use by country and survey year (percentage)

|  | Austria | | Canada | | Israel | | Netherlands | | Romania | | Spain | | Finland | |
| --- | --- | --- | --- | --- | --- | --- | --- | --- | --- | --- | --- | --- | --- | --- |
|  | 2018 | 2020 | 2018 | 2020 | 2018 | 2020 | 2018 | 2020 | 2018 | 2020 | 2018 | 2020 | 2018 | 2020 |
| SMS | 88 | 86 | 70 | 73 | 84 | 80 | 50 | 44 | 58 | 66 | 47 | 48 | 94 | 90 |
| MMS | 28 | 26 | 21 | 26 | 38 | 38 | 5 | 6 | 11 | 16 | 21 | 18 | 55 | 51 |
| Video | 19 | 22 | 13 | 14 | 27 | 33 | 17 | 19 | 18 | 33 | 26 | 26 | 22 | 23 |
| Radio | 13 | 12 | 10 | 9 | 19 | 20 | 11 | 11 | 9 | 11 | 27 | 26 | 16 | 15 |
| Podcast | 1 | 4 | 7 | 7 | 4 | 9 | 1 | 4 | 0 | 2 | 6 | 9 | 3 | 4 |
| Music | 9 | 8 | 13 | 13 | 27 | 28 | 14 | 16 | 15 | 23 | 24 | 26 | 10 | 11 |
| Photos | 87 | 88 | 69 | 68 | 86 | 85 | 78 | 86 | 40 | 56 | 89 | 88 | 80 | 79 |
| Rec Video | 32 | 36 | 25 | 28 | 52 | 57 | 12 | 13 | 20 | 33 | 32 | 34 | 23 | 21 |
| Web Browser | 43 | 50 | 41 | 43 | 63 | 66 | 48 | 52 | 28 | 45 | 54 | 57 | 52 | 53 |
| Web Apps | 37 | 40 | 28 | 33 | 58 | 59 | 45 | 51 | 19 | 32 | 37 | 41 | 29 | 34 |
| Inst mess | 54 | 59 | 18 | 23 | 75 | 82 | 61 | 59 | 28 | 50 | 84 | 85 | 54 | 65 |
| SNS | 26 | 29 | 30 | 31 | 57 | 58 | 37 | 35 | 28 | 40 | 47 | 51 | 33 | 36 |
| Games | 13 | 14 | 20 | 19 | 32 | 28 | 22 | 23 | 12 | 12 | 17 | 18 | 7 | 7 |
| Calendar | 61 | 57 | 45 | 43 | 64 | 59 | 45 | 48 | 26 | 40 | 63 | 59 | 48 | 45 |
| Alarm | 64 | 61 | 45 | 40 | 68 | 64 | 46 | 42 | 32 | 40 | 65 | 62 | 69 | 64 |
| Email | 60 | 63 | 51 | 55 | 75 | 77 | 61 | 66 | 24 | 36 | 73 | 77 | 58 | 63 |
| GPS Maps | 45 | 46 | 32 | 35 | 68 | 65 | 42 | 44 | 17 | 25 | 65 | 62 | 48 | 48 |
| Down. Apps | 38 | 42 | 23 | 27 | 57 | 59 | 50 | 51 | 7 | 14 | 44 | 46 | 25 | 27 |
| Voice calls | 94 | 92 | 78 | 78 | 94 | 94 | 90 | 90 | 70 | 80 | 78 | 80 | 85 | 86 |
| N | 739 | 731 | 411 | 411 | 463 | 454 | 425 | 431 | 497 | 501 | 884 | 882 | 746 | 714 |

Supplementary Table 3. Tetrachoric correlations of different mobile phone functionalities in 2020


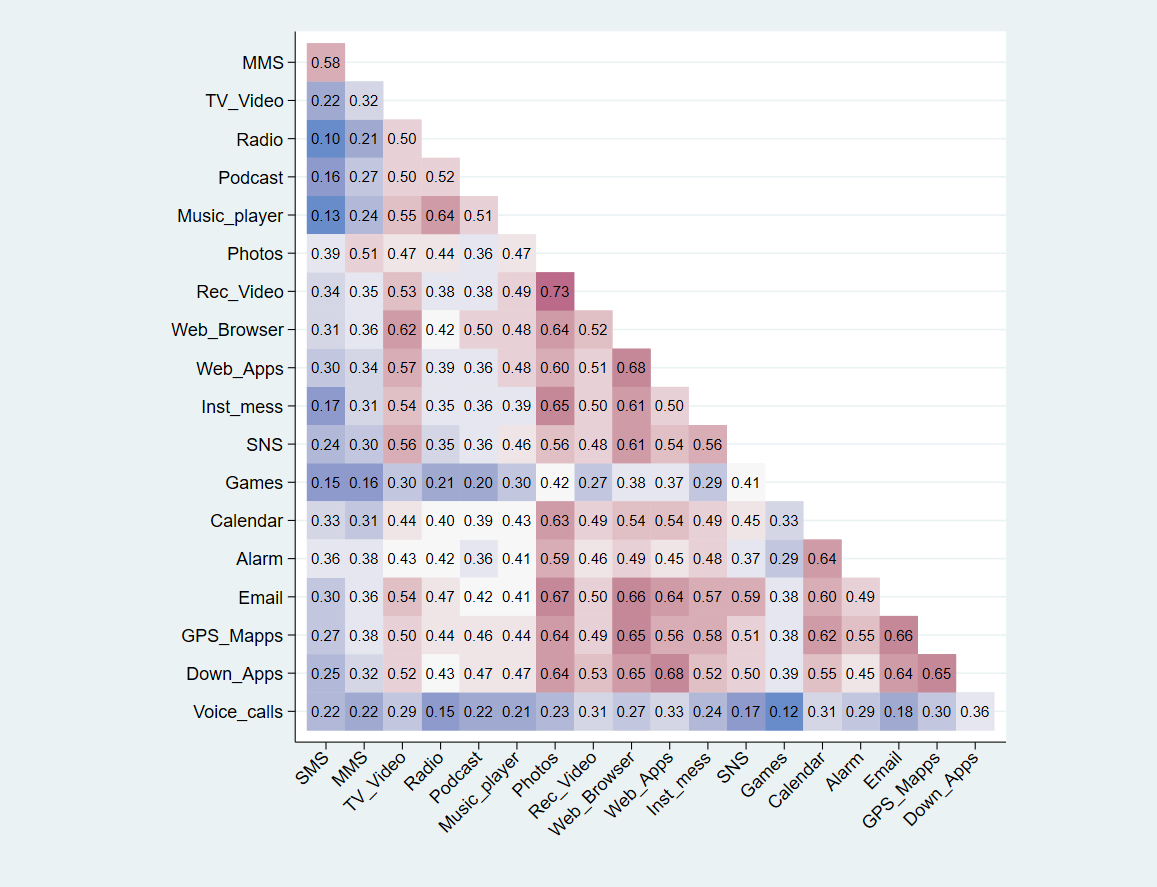


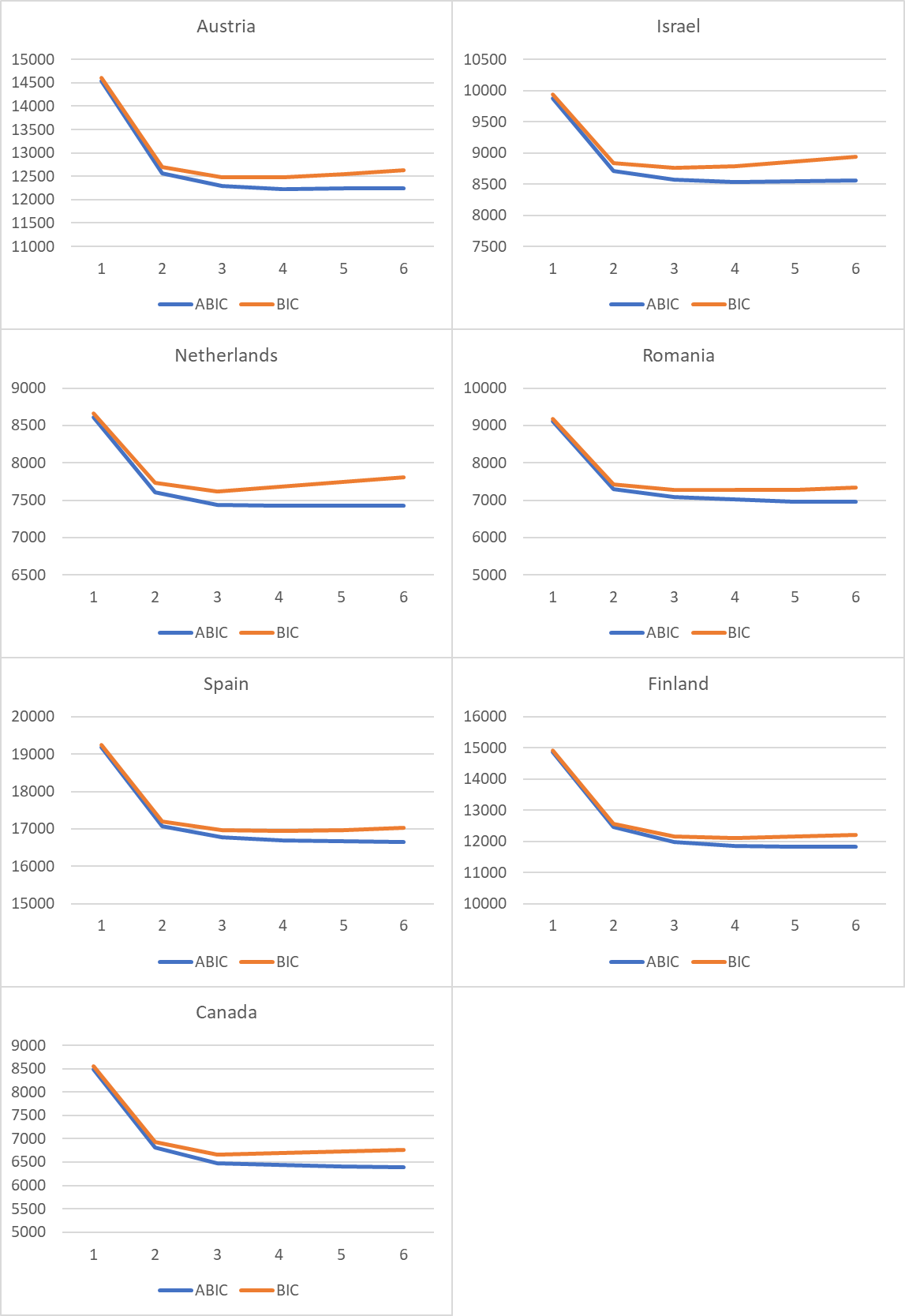


Supplementary Figure 1. Fit indices of different numbers of latent classes by country in 2018 (BIC = Bayesian information criterion; ABIC = sample-size adjusted BIC)


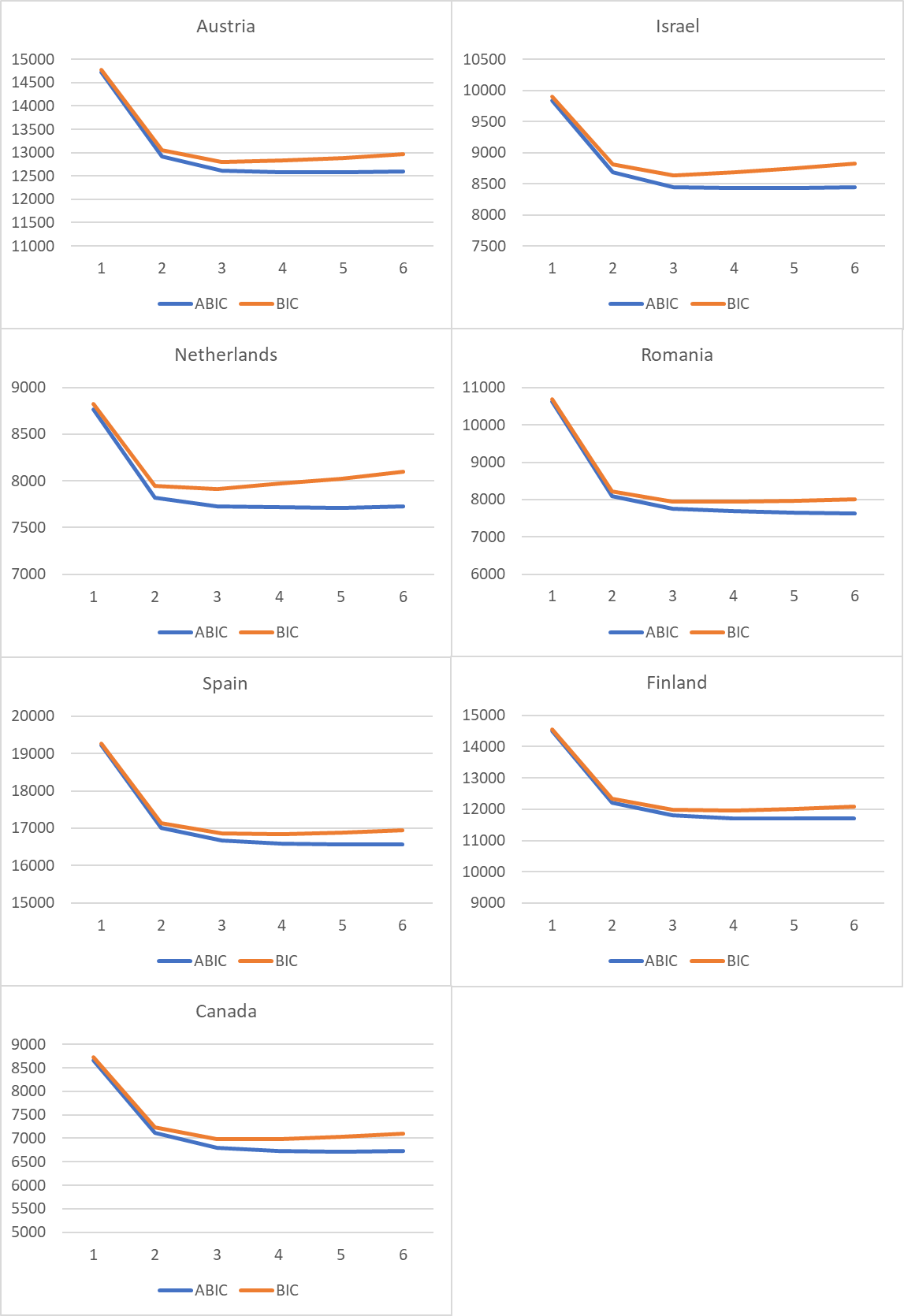


Supplementary Figure 2. Fit indices of different numbers of latent classes by country in 2020 (BIC = Bayesian information criterion; ABIC = sample-size adjusted BIC)
